# Influence of Neighboring Clonal-Colonies on Aflatoxin Production by *Aspergillus flavus*

**DOI:** 10.3389/fmicb.2019.03038

**Published:** 2020-01-15

**Authors:** Rebecca R. Sweany, Kenneth E. Damann

**Affiliations:** Department of Plant Pathology and Crop Physiology, Louisiana State University Agricultural Center, Baton Rouge, LA, United States

**Keywords:** *Aspergillus flavus*, volatile chemical, volatile sensing, aflatoxin, quorum sensing

## Abstract

*Aspergillus flavus* is an ascomycete fungus that infects and contaminates corn, peanuts, cottonseed, and treenuts with acutely toxic and carcinogenic aflatoxins. The ecological function of aflatoxin production is not well understood; though not phytotoxic, aflatoxin may be involved in resisting oxidative stress responses from infection or drought stress in plants. Observation of aflatoxin stimulation in 48-well plates in response to increasing inoculated wells sparked an investigation to determine if *A. flavus* volatiles influence aflatoxin production in neighboring colonies. Experiments controlling several culture conditions demonstrated a stimulation of aflatoxin production with increased well occupancy independent of pH buffer, moisture, or isolate. However, even with all wells inoculated, aflatoxin production was less in interior wells. Only one isolate stimulated aflatoxin production in a large Petri-dish format containing eight small Petri dishes with shared headspace. Other isolates consistently inhibited aflatoxin production when all eight Petri dishes were inoculated with *A. flavus*. No contact between cultures and only shared headspace implied the fungus produced inhibitory and stimulatory gases. Adding activated charcoal between wells and dishes prevented inhibition but not stimulation indicating stimulatory and inhibitory gases are different and/or gas is inhibitory at high concentration and stimulatory at lower concentrations. Characterizing stimulatory and inhibitory effects of gases in *A. flavus* headspace as well as the apparently opposing results in the two systems deserves further investigation. Determining how gases contribute to quorum sensing and communication could facilitate managing or using the gases in modified atmospheres during grain storage to minimize aflatoxin contamination.

## Introduction

*Aspergillus flavus* is an ascomycete fungus that impacts agriculture, public and environmental health due to production of acutely toxic, carcinogenic aflatoxins in oil seed crops (Diener et al., 1987; Wicklow, 1991; Horn, 2003). Aflatoxins are toxic to humans and most animals including mammals, fish, and insects (Diener et al., 1987; Wicklow, 1991; Horn, 2003). Peanuts and corn are especially prone to aflatoxin contamination in tropical and sub-tropical climates (Diener et al., 1987; Wicklow, 1991; Horn, 2003). Aflatoxin production is favored in developing seeds infected with *A. flavus* when crops experience heat and drought stress (Diener et al., 1987; Wicklow, 1991; Horn, 2003).

The ecological function of aflatoxin production in both the soil and phytobiome is not fully understood. Several exogenous factors influence aflatoxin production including: light, pH, water activity, reactive oxygen species (ROS), nitrogen, inorganic, and organic salts etc. (Joffe and Lisker, 1969; Brakhage, 2013; Roze et al., 2013; Woloshuk and Shim, 2013). It is suggested aflatoxin synthesis provides catalase and ROS consumption activity which increases tolerance to oxidative stress from plants during the infection process and under drought conditions (Fountain et al., 2015; Roze et al., 2015). Strains of *A. flavus* without aflatoxin production capabilities (atoxigenic) are also tolerant of H_2_O_2_ oxidative stress and are commonly isolated from plants, demonstrating aflatoxin is not necessary for infection (Diener et al., 1987; Wicklow, 1991; Horn, 2003; Abbas et al., 2005; Giorni et al., 2007; Atehnkeng et al., 2008; Sweany et al., 2011; Fountain et al., 2015) and *A. flavus* may have other mechanisms to limit oxidative stress during plant tissue invasion.

Aflatoxin is toxic to insects and may be important for competition against insects in both the soil and plant ecosystems (Zeng et al., 2006; Rohlfs and Obmann, 2009; Drott et al., 2017). Aflatoxin is toxic to *Drosophila* spp. (Rohlfs and Obmann, 2009; Drott et al., 2017), though some species are less sensitive to aflatoxin (Rohlfs and Obmann, 2009). Greater aflatoxin production in the presence of drosophila larvae and additional growth of both toxigenic and atoxigenic *A. flavus* due to greater larval mortality from supplemental aflatoxin suggest aflatoxin production gives a competitive advantage to the fungus in the enotomopathogenicity vs. fugivory interaction (Drott et al., 2017). In contrast, aflatoxin has limited effects on potential insect vectors that typically occupy similar plant niches as *A. flavus* (Zeng et al., 2006; Drott et al., 2017). Maize weevils are not killed by aflatoxin (Drott et al., 2017). Both toxigenic and atoxigenic *A. flavus* kill corn earworm larvae within a day (Poole and Damann, unpublished data). High concentrations of aflatoxin kill corn earworm larvae (Zeng et al., 2006), but in the presence of several plant compounds larvae produce cytochrome P450 monooxygenases that detoxify aflatoxin (Zeng et al., 2007; Niu et al., 2008). The ecological relationships between insects, aflatoxin production, fungal fitness, entomopathogenicity, and plant are complex with evidence of both toxicity to insects and evolving mechanisms to limit toxicity.

Aflatoxin is speculated to be important for survival in soil microbial communities. Aflatoxin is toxic to some common soil gram+ bacteria including *Bacillus*, *Streptomyces*, and *Nocardia* spp., but has limited toxicity to several fungi and other gram+ and gram− bacteria (Burmeister and Hesseltine, 1966; Arai et al., 1967). Volatiles produced by *Aspergillus nidulans* both stimulate and reduce aflatoxin production in *Aspergillus parasiticus* (Roze et al., 2007). Volatiles emitted from the soil-borne plant pathogenic bacterium *Ralstonia solanacearum* induce chlamydospore production and increase aflatoxin production (Spraker et al., 2014). Intensive research investigated the ability of atoxigenic *A. flavus* strains to competitively exclude toxigenic strains from crops and reduce aflatoxin contamination (Cotty, 1990; Brown et al., 1991; Dorner et al., 1992). Several lines of evidence indicate that there is inhibition of aflatoxin production, but the chemical/biochemical interaction is still not understood (Wicklow, et al. 2003; Mehl and Cotty, 2010; Abbas et al., 2011; Huang et al., 2011). Altering the concentration of conidia in culture modulates aflatoxin production in a density dependent manner similar to quorum sensing in bacteria (Clevström et al., 1983; Brown et al., 2009; Affeldt et al., 2012). At lower concentrations 10^3^ conidia/ml medium, *A. flavus* produces sclerotia, aflatoxin, and limited conidia, in contrast at higher concentrations 10^7^ conidia/ml medium, *A. flavus* shifts from sclerotial to dense conidial production and lower aflatoxin production (Brown et al., 2009; Affeldt et al., 2012). Deletion of oxylipin-generating dioxygenase genes, especially *ppoC*, restores sclerotial production at high inoculum densities and maintains aflatoxin production implicating oxylipins in the quorum sensing developmental shift (Brown et al., 2009; Affeldt et al., 2012). Cessation of aflatoxin production at higher conidial concentrations, suggests that aflatoxin is more important to survival of *A. flavus* at a low population. At low population sizes, there is more competition from surrounding microbes but as the population increases and *A. flavus* successfully colonizes the soil habitat there is less need to produce toxin presumably to compete with other organisms. Alternatively when fewer conidia are present, fewer colonies occupy the same space which results in better hyphal development and colony establishment potentially leading to secondary metabolism and sclerotization.

Initial experiments to determine if there is density-dependent stimulation of aflatoxin production from gases produced by the fungus within 24-well plates found no statistical differences in aflatoxin production if *A. flavus* grew in 6, 12, 18 or 24 wells, though there was a small increase that coincided with an increase in the number of wells with *A. flavus*. However if either 12, 24, 36 or 48 wells were inoculated with a single strain of *A. flavus* in 48-well plates, no aflatoxin was produced when *A. flavus* grew in only 12-wells, but ∼1000, 1500, and 2500 ppb aflatoxin B_1_ was produced if 24, 36 or 48 wells were inoculated, respectively. Therefore, a series of experiments was conducted to determine if there is a density-dependent stimulation of aflatoxin production in *A. flavus*. Evidence is presented that volatile chemicals produced during growth of *A. flavus* both stimulate and inhibit aflatoxin production in a location and density-dependent manner. Understanding the volatile interactions between *A. flavus* has the potential to improve grain storage either by removing stimulatory compounds or modifying the atmosphere with inhibitory gases.

## Materials and Methods

### Fungal Isolates

Several different experiments employed *A. flavus* isolates 53, Tox4 and Af70s. All isolates produce both aflatoxin B_1_ and B_2_, but do not produce any G toxins. Isolate 53 was isolated from corn in Louisiana in 2003 and produces large sclerotia >400 μm (Huang et al., 2011). Tox4 (syn. 07-C-1-1-1) was isolated from corn in 2007, rarely produces large sclerotia and belongs to the same vegetative compatibility group as isolate 53 (Sweany et al., 2011). Af70s was isolated from a cotton field soil in Arizona and produces small sclerotia <400 μm (Cotty, 1989). Tox4 and 53 are both *Mat1-2* and Af70s is *Mat1-1*. All isolates were stored in 50:50 glycerol: water vol/vol at −20°C at Louisiana State University. Af70s was acquired from the USDA-SRRC fungal collection in New Orleans and 53 and Tox4 are deposited in the collection. For each experiment, a fresh batch of conidia grew on 5% V8 agar (pH 5.2) for 5 days and harvested in 2 mL of water by dislodging them with a glass rod.

### Media

Liquid glucose salts (L) medium contained: 3.5 g (NH_4_)_2_SO_4_, 750 mg KH_2_PO_4_, 350 mg MgSO_4_⋅7H_2_O, 75 mg CaCl_2_⋅2H_2_O, 10 mg ZnSO_4_⋅7H_2_O, 5 mg MnCl_2_⋅4H_2_O, 2 mg NH_4_Mo_7_O_24_⋅4H_2_O, 2 mg Na_2_B_4_O_7_⋅10H_2_O and 2 mg FeSO_4_⋅7H_2_O and 50 g D-glucose per liter (Wicklow et al., 2003). The salts solution and glucose were autoclaved separately. Citrate buffer (0.053 M citric acid and 0.027 M sodium citrate) was added to medium (L-buffered) to buffer at pH4, maximize aflatoxin production, minimize degradation and prevent extreme acidification of medium to less than 2 (Doyle and Marth, 1978a, b; Cotty, 1988). Agar (2% wt/vol) was added to either non-buffered (S) or buffered (S-buffered) glucose salts medium.

### Examination of *A. flavus* Well Occupancy Effects on Aflatoxin Production

A series of experiments investigated stimulation and inhibition of aflatoxin production by gases produced from increasing the number of *A. flavus* cultures in 48-well plates (a closed system). To rule out possibilities other than gaseous production by *A. flavus*, culture conditions were manipulated within 48-well plates.

**FIGURE 1:**
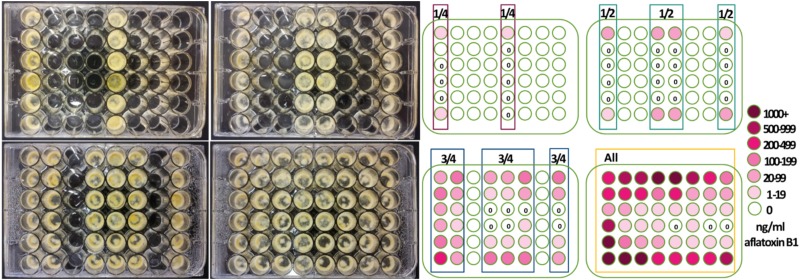
Number of inoculated wells and well-location changed aflatoxin production by *Aspergillus flavus*. 48-well plates were inoculated with a single isolate 53 of *A. flavus* in the four arrays depicted. Centrally located wells produced more conidia and growth appeared less dense when all wells were inoculated. Aflatoxin production was less in the interior wells and greatest when all wells were inoculated with *A. flavus*.

**(A)** Initial experimental design (Figure 1). To determine if the number of cultures within a closed system alters aflatoxin production of an individual strain, within four Costar flat bottom 48-well plates (3548 Corning, New York, NY, United States) either 12, 24, 36 or 48 wells were filled with 500 μl 1 × 10^5^ *A. flavus* 53 conidia/ml L-buffered glucose-salts medium; the other wells remained empty. 48-well plates consisted of 8 columns with six wells. All wells within either columns 1 and 5; 1, 4, 5, and 8; 1, 2, 4, 5, 6, and 8 or all 8 columns were filled with inoculated medium. Fungal colonies within wells were considered independent samples because the fungus and medium did not have contact with other wells and therefore should be independent resulting in either 12, 24, 36 or 48 replicated samples per plate. Individual wells (11.3 mm diameter) were attached to neighboring wells but separated by well walls with 1 mm thickness. The 48-well plates were wrapped in Parafilm, placed in individual boxes atop two wet paper towels and incubated for 4 days in the dark at 25°C. The fungus produced hyphal mats that primarily grew 0–3 mm on and below the medium-air interface. Aflatoxin was extracted from individual wells by mixing 240 μl of remaining medium below the hyphal mat with 240 μl acetonitrile. Aflatoxin B_1_ was quantified with HPLC as described below. To determine if the differences in aflatoxin production were due to gases produced by the fungus or culture conditions, several questions were investigated as follows:

**(B)** Does the lack of fungus and/or medium in empty wells cause the changes in aflatoxin production, i.e., do volatiles released from additional medium or fungal growth change aflatoxin production? Since there were empty wells in the initial experimental design, all non-inoculated wells were filled with L-buffered medium. As in the first experiment, either 12, 24, 36 or 48 wells were filled with 500 μl 1 × 10^5^ *A. flavus* 53 conidia/ml L-buffered medium, but now the remaining wells were filled with L-buffered medium. Plates were incubated for 4 days and aflatoxin extracted as described above. Aflatoxin B_1_ was quantified by HPLC as described below.

**(C and D)** Does solidified medium minimize the differences between wells? Agar was added to the medium to minimize 20–40 μl moisture loss in exterior wells and determine if differences in aflatoxin production were in response to more fungal growth within a closed system or moisture loss. All wells were filled with 500 μl of either S-buffered or non-buffered S-medium. As described above, either 12, 24, 36 or 48 wells were center-point inoculated with 1.25 × 10^7^ *A. flavus* 53 conidia/ml in 1 μl. Plates were incubated for 4 days as above. To extract aflatoxin, medium and spores from each well were placed in a screwtop 3.7 ml glass vial filled with chloroform and incubated overnight in a fume hood. The chloroform was filtered through Whatman filter paper and evaporated overnight. The aflatoxin was resuspended in 0.5 ml of 80:20 vol/vol methanol: water and then mixed with 0.5 ml acetonitrile. Aflatoxin B_1_ was quantified by HPLC as described below.

**(E and D)** Are the differences in aflatoxin production maintained without citrate pH buffer? Since citric acid is an important component of metabolism (Buchanan et al., 1985), 53 was grown in both S and L non-buffered medium. As described in (A), *A. flavus* suspended in liquid glucose salts medium was grown in 12, 24, 36 or 48 wells of 4–48 well plates without medium in non-inoculated wells. Additionally, all wells of 5th 48-well plate were filled with L-medium but only 12 wells contained conidia. The design for non-buffered S-medium was described in (C and D). Plates were incubated for 4 days and aflatoxin extracted as described above. Aflatoxin B_1_ was quantified by HPLC as described below.

**(F)** Does reducing airflow with Parafilm remove differences between wells? To reduce exchange or release of chemicals from individual wells, all wells of a 48-well plate where filled with 500 μl 1 × 10^5^ *A. flavus* 53 conidia/ml L-buffered medium. Four layers of Parafilm was placed flush atop the wells, the lid placed on top, wrapped in Parafilm, incubated for 4 days and aflatoxin extracted as described above. Aflatoxin B_1_ was quantified by HPLC as described below.

**(G)** Does adsorbing gases to activated charcoal remove differences between wells? Activated charcoal was pipetted in the spaces between the wells filled with buffered L-medium. As in the second experiment, either 12, 24, 36 or 48 wells were filled with 500 μl 1 × 10^5^ *A. flavus* 53 conidia/ml L-buffered medium; the remaining wells were filled with L-buffered medium. Plates were incubated for 4 days and aflatoxin extracted as described above. Aflatoxin B_1_ was quantified by HPLC as described below.

**(H and I)** Does changing the isolate change the influence of the number of cultures on aflatoxin production? To determine if alteration in aflatoxin production due to changing the number of wells with *A. flavus* was isolate specific; a second isolate, Af70s was used. Either 12, 24, 36 or 48 wells were filled with 500 μl 1 × 10^5^ *A. flavus* Af70s conidia/ml L-buffered medium; wells without conidia were either empty (H) or filled with L-buffed medium (I). Plates were incubated for 4 days and aflatoxin extracted as described above. Aflatoxin B_1_ was quantified by HPLC as described below.

#### Rationale for Staggered Experiments

The above experiments were conducted serially due to incubator and sample processing constraints. The incubator could easily accommodate four plates in individual boxes and up to eight plates. Four plates consisted of 120 samples. Aflatoxin B_1_ slowly degraded in the samples. To avoid confounding effect of aflatoxin degradation, only single variables were manipulated at a given time and compared to the first experiment A. The HPLC machine became irrepairable, therefore experiments in 48-well plates were not repeated and changing to a different pH buffer was not possible. Experiment A was repeated and the results were similar.

### Examination of *A. flavus* Petri-Dish Occupancy on Aflatoxin Production

Though the chances of chemical diffusion between the wells is low, independent interior wells within 48-well have 4-(2 mm deep by 3 mm long) junction points. A method employed to investigate the influence of volatile production by *A. nidulans* on *A. parasiticus* was modified (Roze et al., 2007) to determine if gases produced by *A. flavus* grown in separate Petri-dishes with shared headspace affect aflatoxin production. Eight open 35 mm wide small Petri-dishes (Falcon 351058, Corning, New York, NY, United States) were nestled within a larger 150 mm diameter Petri-dish separated by ∼2 mm. All small Petri-dishes were filled with 4 ml of non-buffered S-medium. Either 1, 4 or 8 plates were center point inoculated with 1 μl of 1 × 10^8^ conidia/ml of a single *A. flavus* isolate and the large dish was wrapped in two layers of Parafilm. Each inoculated dish was considered an independent sample and the experiment was replicated three times resulting in either 3, 12 or 24 replicated samples per condition. The dishes were incubated for 5 days at 25°C in the dark. Initially only isolate 53 was used. Aflatoxin was extracted from each plate by combining solid medium and spores in a 20 ml scintillation vial with chloroform and incubated overnight in a fume hood. The chloroform was filtered through Whatman filter paper and evaporated overnight. The aflatoxin was resuspended in 0.5 ml of 80:20 vol/vol methanol: water and then mixed with 0.5 ml acetonitrile. Aflatoxin B_1_ was quantified by HPLC as described below.

Three grams of activated charcoal was added to the bottom of the large Petri dishes to adsorb volatile gases produced by *A. flavus*. Three different isolates (53, Tox 4 and Af70s) were independently center-point pipetted onto either 1, 4 or 8 plates with and without activated charcoal added to the base of the large dish and all treatments were replicated three times. Dishes were incubated for 5 days and aflatoxin extracted as described above. Aflatoxin B_1_ was quantified by HPLC as described below.

### Aflatoxin B_1_ Quantification

All extracts were filtered through 1.5 ml polypropylene columns with 20 μm polyethylene frits, packed with 200 mg basic aluminum oxide (58Å, 60-mesh powder, 11503-A1, Alfa Aesar, Tewksbury, MA, United States) into an auto-sampler vial (Sobolev and Dorner, 2002). The aflatoxin was quantified with reversed-phase high performance liquid chromatography using a Summit HPLC System (Dionex Corporation, Sunnyvale, CA, United States) with a P580 pump, ASI-100 automated sample injector, RF2000 fluorescence detector, and Chromeleon software version 6.20 (Joshua, 1993). A post-column derivatization step was conducted by exposing the extract to a UV light in a PHRED cell (Aura Industries Inc., New York, NY, United States) (Joshua, 1993). The mobile phase was 22.5 parts HPLC grade methanol: 22.5 parts HPLC grade acetonitrile: 55 parts distilled water mixture at 1 ml/min. The stationary phase was a Syncronis C18, 3 × 150 mm long column (Thermo Fisher Scientific Inc., Waltham, MA, United States). Aflatoxin B_1_ was detected at 11.8 min and quantified by Chromeleon software using to pure 1, 10, 100, and 1000 ng aflatoxin B_1_/ml standards.

### Data Analysis

Aflatoxin B_1_ means and standard error were calculated using Excel (Microsoft Corp., Redmond, WA, United States). Statistical analysis was conducted using SAS version 9.4 (SAS Institute, Cary, NC, United States). In the 48-well plates, individual wells were considered the sampling unit; *n* = 1020 across all individual experiments (including the different isolates). To partition the variance and account for heterogeneity within single 48-well plates, wells belonging to rows A and F (outter-most), B and E (intermediate) or C and D (inner-most) were treated as reps (4 reps/row location for 12 inoculated wells, 8 reps for 24, 12 reps for 36 and 16 reps for 48 inoculated wells within individual 48-well plates). Linear models approximated MANOVAs using proc Mixed. For the 48-well plate experiments, three full models were evaluated. The first model assessed the fixed categorical effects: media (L and S-medium and L and S- buffered medium), isolate (53 and Af70s), well location (rows A and F, B and E, and C and D), and number of inoculated wells (12, 24, 36 or 48) on log (aflatoxin concentration +1). A second full model assessed the fixed categorical effects activated charcoal, number of inoculated wells and location on log (aflatoxin concentration +1). The final full model tested the fixed categorical effects of Parafilm and location on aflatoxin concentration. Interactions between fixed effects were statistically significant if the *p*-value of *F*-test for type III fixed effects was less than 0.05. Due to significant interaction terms of full models, separate MANOVAs were calculated for each of the 48-well plate experiments described above (A–I) with fixed effects of well location and number of inoculated wells on log (aflatoxin +1). For the Petri-dish design two models were conducted. Individual Petri dishes were considered the sampling unit and each experiment was replicated three times. An initial model for isolate 53 accessed the fixed effects of location and number of inoculated dishes (1, 4 or 8) on aflatoxin concentration (*n* = 39). A second full model assessed the fixed effects of number of inoculated plates, strains (Tox4, 53 and Af70s) and activated charcoal on aflatoxin (*n* = 234). Due to significant interaction terms, separate MANOVAs by isolate were calculated. *Post hoc* comparison of means were calculated using Tukey-Kramer adjusted means and considered different if the *p*-value was 0.05 or less. Where appropriate, aflatoxin values were adjusted to log (toxin +1) to meet the assumption of normality.

## Results

### Neighbors and Well-Location Alter Aflatoxin Production

*Aspergillus flavus* colony morphologies and aflatoxin production differed both when the number of neighboring wells within 48-well plates filled with isolate 53 conidia (in liquid buffered glucose salts medium i.e., L-buffered medium) and well-location changed (Figure 1). Mycelial and conidial production appeared less dense when *A. flavus* grew in all 48 wells especially in comparison to growth of *A. flavus* in 12 wells. This contrasted with a stimulation of aflatoxin production (Table 1, A). Increasing the number of wells with *A. flavus* stimulated aflatoxin production by as much as 900 ppb produced if *A. flavus* grew in all 48 vs. 12-wells. Regardless if grown in 12 to 48 wells, *A. flavus* in centrally located wells produced more conidia. In contrast to gain of conidial production, aflatoxin production was inhibited in the interior wells.

**TABLE 1:** Variable aflatoxin production within and between single isolates grown in individual wells of 48-well plates.

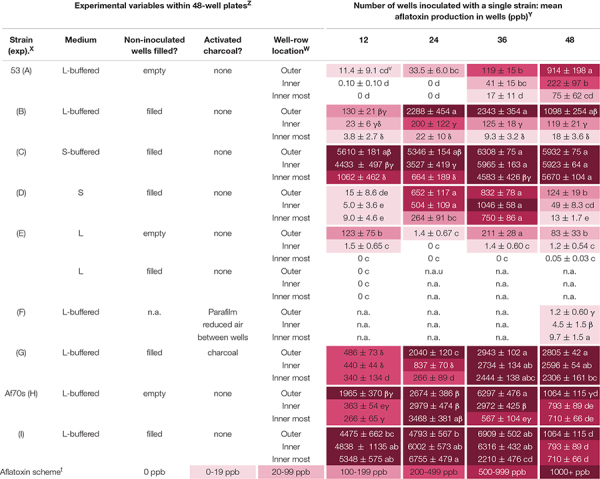

*To determine if increasing number of *A. flavus* cultures stimulates aflatoxin production, aflatoxin was extracted from individual wells of 48-well plates with variable number of identical *A. flavus* cultures. Plates incubated separately in boxes for 4 days in the dark at 25°C. ^*z*^ Several different variables were manipulated to determine consistency of aflatoxin differences between wells in 48-well plates, including: isolates (53 and Af70s), media [(liquid (L) vs. solid (S) glucose salts medium), (citrate buffered vs. non-buffered glucose salts medium)], medium-filled vs. empty non-inoculated wells, activated charcoal between wells, and row (six total) location of cultures within plates. ^*y*^ To determine the influence of the number of cultures on aflatoxin production within 48-well plates, *A. flavus* conidia were pipetted into all wells of columns 1 and 5 (1/4 plate or 12 wells); 1, 4, 5, and 9 (1/2 plate or 24 wells); 1, 2, 4, 5, 6, and 8 (3/4 plate or 36 wells) or all 48 wells of the plate. ^*x*^ Individual experiments denoted by capitalized letters investigated several different questions: (A) Does number of identical *A. flavus* cultures within 48-well plates alter aflatoxin production? (B) Is heterogeneity in aflatoxin production caused by lack of medium in the empty wells or more *A. flavus* growth? (C) and (D) Does solidified medium minimize the differences between wells? (E) and (D) Are the differences in aflatoxin production maintained without citrate pH buffer? (F) Does reducing air-flow with Parafilm remove differences between wells? (G) Does adsorbing gases to activated charcoal remove differences between wells? (H) and (I) Is isolate Af70s aflatoxin production influenced by the number of cultures? ^*w*^ Well location refers to the row a well belonged to within an individual 48-well plate. The outer rows are closest to the exterior of the plate (rows A and F), inner rows are the next rows inward from the edge (rows B and E) and the inner-most rows are in the middle of the plate (rows C and D). ^*v*^ Different letters indicate different mean aflatoxin B_1_ ppb ± standard error values based Tukey-Adjusted Least Significant Differences at α < 0.05. For simplification, differences of means are only reported within an individual experiment and not across experiments. Letters alternated from the English to Greek alphabet between individual experiments. ^*u*^ n.a. means not applicable because no experiment conducted. ^*t*^ Colors of the cells represent a heat map of aflatoxin production the colors are the same in Figure 1. Values above 1000 ppb are the same color, therefore, variation in exp. (C) and (I) are not visible.*

### Filling Empty Wells Does Not Alter Location and Neighbor Effect

Overall, filling the non-inoculated wells with medium resulted in higher levels of aflatoxin production, which was most pronounced in the outer wells (Table 1, B). Regardless, inoculating more wells with *A. flavus* still stimulated aflatoxin production by at least 2000 ppb if more than 12 wells were inoculated, suggesting volatiles from both the identical fungal cultures and buffered liquid medium stimulated aflatoxin production. The addition of medium did not change the location effect; aflatoxin production was inhibited substantially within the interior wells, suggesting volatiles from the fungus (not additional medium) also inhibited aflatoxin production within the center of the plates.

### Solid Medium Can Minimize Location and Neighbor Effect

Overall, *A. flavus* produced more aflatoxin on solidified medium. Growth on S-buffered medium minimized the location effect when all wells were inoculated, but not when 12 or 24 were inoculated (Table 1, C & D). The location effect was still pronounced on non-buffered S-medium, with the exception of 36 inoculated wells. Increasing the number of inoculated wells on both S-media stimulated aflatoxin production. On S-buffered medium, more inoculated wells resulted in a minimal increase in aflatoxin production, though in the innermost wells there was still a trend for increasing aflatoxin production. On non-buffered S-medium, there was an overall increase of 865 ppb as the number of inoculated wells increased from 12 to 36, followed by a decrease when all 48 wells were inoculated. Controlling for moisture loss with agar did not completely remove interior inhibition and stimulation by neighbors which suggests fungal volatiles also contribute to these effects.

### Buffering pH Enhances the Response to Neighbors but Not Location

Removing the citrate pH buffers decreased the sensitivity to neighboring colonies only in L-medium and did not change the location effect. The pH decreased from 4 to 2 in the non-buffered L-medium, which coincided with less aflatoxin, sparse mycelia and conidial production compared to buffered L-medium. There was an inconsistent aflatoxin response to the number of wells inoculated with *A. flavus* in liquid medium (Table 1, E). Regardless, the highest aflatoxin production within an individual well (473 ppb) occurred when all 48 wells were inoculated. On S-medium (Table 1, D), removing pH buffers did not alter the stimulatory effect of neighboring cultures. Aflatoxin production was smallest in the interior of plates on both non-buffered media.

### Minimizing Gas Exchange Limits Differences and Aflatoxin Production

Aflatoxin levels were essentially zero in all the wells when 4-layers of Parafilm were placed across the tops of the wells to reduce air exchange (Table 1, F). This was a stark contrast to 900 ppb aflatoxin production in the exterior rows with no Parafilm. In addition to minimal aflatoxin production, the location effect was lost, and when Parafilm was used there was slightly more aflatoxin production in the interior wells. Limiting gas exchange between wells removed both the stimulation caused by more inoculated wells and the inhibition in the interior of the plate.

### Activated Charcoal Removes Inhibitory Gas(es)

Addition of activated charcoal between the wells of 48-well plates to adsorb volatile compounds resulted in an overall increase in aflatoxin production (Table 1, G). Regardless of stimulation by volatile adsorption, aflatoxin was further stimulated if *A. flavus* grew in more than 24 wells. With the exception of 24 inoculated wells, there was no longer a significant loss of aflatoxin production in the interior wells of the 48-well plates. The inhibition of aflatoxin production in the interior of the plate was 30% (12 wells), 90% (24 wells), 17% (36 wells), and 18% (48 wells) when activated charcoal was placed in between the wells, whereas if no activated charcoal was added the inhibition in the interior was 97, 99, 99, and 98% indicating the activated charcoal removed an inhibitory volatile compound.

### Neighbors and Location Also Affect Af70s

A second isolate Af70s increased aflatoxin production as the number of inoculated wells increased from 12 to 36 wells by 2–4000 ppb (Table 1, H & I) regardless of whether the non-inoculated wells were empty or filled with buffered L-medium. Inoculation of all wells resulted in a reduction in aflatoxin production. Af70s produced more aflatoxin than isolate 53. The location effect changed for Af70s. Aflatoxin production increased in the interior of the plate when 12 or 24 wells were inoculated, but decreased in the interior of the plate when 36 and 48 wells were inoculated. More inoculated wells stimulated aflatoxin production regardless of isolate, but the location effect was different between strains.

### Location Effect Is Design Dependent

**FIGURE 2:**
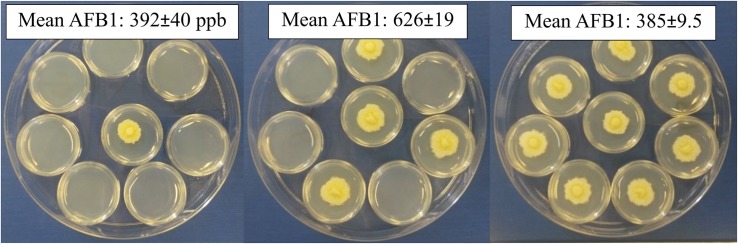
Changing the number of inoculated open Petri-dishes within a closed dish alters aflatoxin production. Either 1, 4 or 8 small Petri-dishes were center-point inoculated and enclosed in a larger dish. All produced similar colonies with dense hyphal growth and minimal conidia; occasionally aerial hyphae could be observed for those with eight inoculated dishes.

Neighboring wells are actually attached; there was a chance chemicals can permeate and pass between the polystyrene wells, though not likely due to 2 mm thick plastic at the junction points. A second experimental design did not allow the culture containers (small Petri dishes) to touch physically (Figure 2). In the previous design, cultures were separated by 2 mm and each culture completely filled the 11.3 mm diameter wells. The second design, 35 mm Petri dishes were separated by only 1–2 mm and colony diameters were approximately 20 mm after 5 days growth. Aflatoxin production increased as the number of dishes with isolate 53 increased from 1 to 4 by 200 ppb, followed by a decrease when eight dishes were inoculated, indicating the stimulatory and inhibitory effects were likely a response to gases emitted from the fungus (Figure 2). Unlike within the 48-well plates, Petri dish location did not significantly alter aflatoxin production, although there were fewer locations to analyze (*p* = 0.592).

Adding activated charcoal to the larger Petri-dish significantly increased aflatoxin production by at least 1000 ppb (Table 2). Unlike the previous experiment, there was only a marginal increase (75 ppb) in aflatoxin when four compared to one dish inoculated with isolate 53 and no activated charcoal used, but aflatoxin production still significantly decreased when all eight dishes were inoculated. Aflatoxin production if eight dishes were inoculated was restored to same amount as if one or four dishes were inoculated when activated charcoal was added to the large Petri-dish, suggesting an inhibitory volatile compound produced by the fungus was adsorbed by the activated charcoal.

**TABLE 2:** Variable aflatoxin production in 8-Petri-dish system.

Strain^z^	Activated charcoal?^y^	Number dishes inoculated with a single strain: mean aflatoxin production/dish (ppb)		
		1^x^	4	8
53	none	1225 ± 274 cd	1299 ± 80 d	587 ± 23 e
	charcoal	3476 ± 98 a	2440 ± 119 b	1689 ± 63 c
Af70s	none	3481 ± 511 αβ	2464 ± 131 βγ	1579 ± 105 δ
	charcoal	2988 ± 462 αβγ	3565 ± 152 α	2078 ± 159 γδ
Tox4	none	2707 ± 180 ab	1675 ± 144 c	763 ± 49 d
	charcoal	3331 ± 743 a	2401 ± 138 b	1312 ± 84 c

*Different number of eight small Petri-dishes were center point inoculated with the same *A. flavus* isolates and sealed with in a larger Petri-dish to determine if aflatoxin production changed if more cultures grew near one another. ^*z*^ Three different isolates were tested to determine if each responded similarly. ^*y*^ Activated charcoal was added to the bottom of the large Petri-dish to adsorb gases produced by *A. flavus*. ^*x*^ Different letters indicate different mean aflatoxin B_1_ ppb ± standard error values based Tukey-Adjusted Least Significant Differences of less than α < 0.05. For simplification, differences of means are only reported across rows (number of dishes inoculated) and between columns (activated charcoal added) within an individual isolate and not across isolates. Letters alternated from the English to Greek alphabet between isolates.*

Aflatoxin production decreased for Tox4 and Af70s as more dishes were inoculated with the fungus (Table 2). Both Tox4 and Af70s produced more aflatoxin than 53 with Af70s producing the most aflatoxin. For isolates Af70s and Tox4, adding activated charcoal resulted in an increase in aflatoxin and restored the aflatoxin production to the same amount as if three or four fewer plates were inoculated. Unlike 53 and Tox4, Af70s produced less, though not statistically significant, aflatoxin when activated charcoal was applied to the large dish with only one inoculated dish.

### Common Themes

Across all the experiments in 48-well plates, inoculating more wells with *A. flavus* isolate 53 increased aflatoxin production consistently. The increase was independent of pH buffering, solidified vs. liquid medium and adsorption of volatile compounds by activated charcoal. Only preventing air movement between the wells with Parafilm lowered aflatoxin production. Inoculating more wells also stimulated aflatoxin production for isolate Af70s, but inoculating all 48 wells decreased aflatoxin production. The stimulation was isolate independent, in spite of the final inhibition. In the Petri-dishes, isolate 53 also stimulated aflatoxin production as the number of inoculated dishes increased, but inoculating all eight dishes inhibited aflatoxin production. Isolates Tox4 and Af70s only inhibited aflatoxin if more dishes were inoculated. For each strain, the addition of activated charcoal resulted in an increase in aflatoxin comparable to having three or four fewer inoculated Petri-dishes, suggesting the adsorption of an inhibitory volatile organic compound produced by the fungi.

The location of a well within 48-well plates changed the amount of aflatoxin production. Growth in the center of 48-well plates consistently reduced aflatoxin production independent of pH-buffer for strain 53. Addition of activated charcoal minimized differences between interior and exterior wells, suggesting the adsorption of inhibitory volatile compounds. Addition of agar to buffered medium (not filling empty wells with liquid buffered medium) also minimized the location effect, suggesting an interaction between matric potential and buffering capacity on inhibition. Location of isolate Af70s in the 48-well plates had a different effect on aflatoxin production. Inoculating a few wells increased aflatoxin production in the inner wells, but inoculating more wells decreased aflatoxin production in the inner wells. Location of the dishes within a larger Petri-dish did not affect aflatoxin production and this was consistent between strains.

## Discussion

Neighboring colonies (or cultures) of identical *A. flavus* isolates grown within a closed system altered the aflatoxin production of one another in the absence of physical contact. Different responses occurred, either inhibition and or stimulation of aflatoxin production, which were attributable to growth of other *A. flavus* cultures, not culture conditions. By controlling different variables, results suggested different volatile organic compounds or inorganic gases likely contributed to the inhibition and stimulation of aflatoxin production. Understanding how these volatile interactions influence aflatoxin regulation, competition by *A. flavus* and aflatoxin accumulation both pre and post-harvest deserves further attention.

In general, the number of independent cultures of a single *A. flavus* isolate grown within a closed system altered the aflatoxin production within physically separated wells or petri-dishes. This was an interaction between genetically identical *A. flavus* changing aflatoxin production with only a shared headspace and without direct physical interaction or sharing the same medium suggesting heterogeneity in the gaseous environment from fungal growth altered aflatoxin production. Conidial concentration within either liquid or solid cultures regulates aflatoxin production in single *A. parasiticus* and *A. flavus* isolates, usually aflatoxin production decreases with increased concentration (Clevström et al., 1983; Brown et al., 2009; Affeldt et al., 2012). The decrease in aflatoxin production is associated with a developmental shift away from sclerotial production [sclerotia can serve as stromata for sexual ascospore production (Horn et al., 2009; Horn et al., 2016)] to increased asexual conidial production (Brown et al., 2009; Affeldt et al., 2012). This is similar to the inhibition observed by increasing the number of identical *A. flavus* cultures growing on small Petri-dishes with a shared headspace or the increase in culture density experienced in the center of 48-well plates, though there was only observed changes in conidial or sclerotial production within 48-well plates. In contrast, herein is reported a novel increase in aflatoxin production and decrease in conidia in response to increasing number of identical *A. flavus* cultures from 12 to 48 within 48-well plates with only shared headspace (i.e., more conidia in same space). Atoxigenic *A. flavus* can inhibit aflatoxin production if grown in the same medium (including corn) but only when there is direct contact with toxigenic isolates (Wicklow et al., 2003; Mehl and Cotty, 2010; Abbas et al., 2011; Huang et al., 2011). Neighboring Petri-dish cultures of both *A. nidulans* and *R. solanacearum* that share headspace with *A. parasiticus* and *A. flavus* affect aflatoxin production (Roze et al., 2007; Spraker et al., 2014). In closely related *A. parasiticus*, an increase in the number of Petri-dishes inoculated with *A. nidulans* inhibits aflatoxin production and conidiation of *A. parasiticus* within a closed larger Petri-dish (Roze et al., 2007). The inhibition is attributed to *A. nidulans*, but there is also a decrease in the number of dishes inoculated with *A. parasiticus*, perhaps some of the inhibition in toxin production could have been due to a lack of stimulation from neighbors of the same species (Roze et al., 2007). *R. solanacearum* grown in separate petri-dishes increases aflatoxin production in *A. flavus* (Spraker et al., 2014). Aflatoxin production is regulated by chemicals produced in media and released as gases from other members of the same isolate, other isolates of the same species, other *Aspergilli* and bacteria.

Culture-system design and to a lesser extent isolate altered the aflatoxin production response to *A. flavus* growing in close proximity to one another. On Petri-dishes mostly one phenomenon was observed, an inhibition of aflatoxin production as the number of cultures increased. In contrast, two contradictory phenomena were observed in 48-well plates. Increasing the number of cultures stimulated aflatoxin production, whereas cultures in the center of the plate inhibited aflatoxin production. The difference between wells in the center of 48-well plates vs. outer rows is similar to an edge effect, suggesting a gradient of volatile chemicals can alter the aflatoxin production potential of a colony. It would have been expected if volatiles from neighboring cultures stimulate aflatoxin production, both a stimulation from increasing the number of inoculated wells and in the center of plates (where extensively there is an increase in well density and build up of chemicals). Potentially those gases that are stimulatory become inhibitory at higher concentrations or there are both stimulatory and inhibitory gases produced and stoichiometrically more inhibitory gas molecules are produced or the fungus is more sensitive to the inhibitory gases. There were also two other seemingly contradictory phenomena, when non-inoculated wells were filled with liquid medium or agar was added to medium to control moisture loss, only solidified buffered medium minimized the location effect and number of culture effect. Potentially, adding agar not only controlled moisture loss, but also changed the matric potential of the medium and affected the ability of gases to be adsorbed into the medium and therefore minimized the effect of volatile gases on fungal growth. The different responses of 53, Af70s and Tox4 to neighbors is not surprising, since aflatoxin production, biocontrol capacity and volatile production varies among isolates (Cotty, 1989; Zeringue et al., 1993; Mehl and Cotty, 2010; Abbas et al., 2011; Huang et al., 2011; De Lucca et al., 2012). It deserves further review to determine if mixing isolates within 48-well plates or Petri-dishes alters aflatoxin production especially to determine if atoxigenic isolates’ volatiles can inhibit or stimulate aflatoxin production. Altering culture system design can alter aflatoxin production; *A. flavus* produces much less aflatoxin on bottom of stacks of dishes filled with peanuts (Xue et al., 2003). Spreading dishes on trays and adding spacers between trays and rotating trays during incubation ameliorates the location effect (Xue et al., 2003). All together these results suggest when screening for anti-fungal and anti-aflatoxin potential of compounds and fungi or genetic resistance it is essential to tailor culture systems that maximize phenotypic homogeneity among *A. flavus* cultures and minimizes the interaction caused by changes in the gaseous environment from fungal growth.

Since activated charcoal adsorbs both inorganic and organic gases, and adding activated charcoal resulted in greater aflatoxin production, it is likely *A. flavus* produced inhibitory volatile organic or inorganic compounds (Smíšek and Černý, 1967). The addition of activated charcoal to the bottom of Petri-dishes resulted in higher aflatoxin production and removed the inhibition from adding more cultures within the larger Petri-dishes. Activated charcoal also minimized the reduction of aflatoxin in the center of the 48-well plates. This suggests the same volatile chemicals could be responsible for the aflatoxin reduction in the 48-well plates and Petri-dishes. If the volatile chemicals are organic they may be related to the oxylipins or psi factors produced by *A. flavus* and *A. parasiticus* that have been reported to be responsible for quorum sensing and reduction of aflatoxin with increased inoculum concentrations (Brown et al., 2009; Affeldt et al., 2012). It is unknown if psi factors are volatile chemicals, but they are in the same biosynthetic pathway as methyl jasmonate, which may suggest volatility (Affeldt et al., 2012). In the interior rows of the 48-well plates, there was typically more conidial production and those conidia were greener than the outer rows, which is consistent with the psi-factor quorum sensing phenotype changes (Brown et al., 2009; Affeldt et al., 2012). Other possible organic volatiles have been demonstrated to lower aflatoxin production in *A. flavus* and *A. parasiticus* include: ethylene (from *A. nidulans* and *A. parasiticus*), *trans*-2-hexanol (from soybean), and at high concentrations 2-buten-1-ol (from *A. nidulans*) (Roze et al., 2004, 2007; De Lucca et al., 2011). Inorganic CO_2_, H_2_O, and O_2_ gases involved in respiration would likely be adsorbed to activated charcoal. High levels of CO_2_ (20%+) have been demonstrated to dramatically lower aflatoxin production, but lower levels at 3% can be a stimulatory (Davis and Diener, 1968; Sanders et al., 1968; Roze et al., 2004). In addition, reduction in O_2_ below atmospheric levels can reduce aflatoxin production but the magnitude is not as great as the increase in CO_2_ (Davis and Diener, 1968; Clevström et al., 1983; Ellis et al., 1993). The effect of H_2_O, either as buildup of free moisture or moisture loss should have been controlled by filling non-inoculated wells or using solidified medium, respectively. There was more aflatoxin production on solidified medium, but there was still inhibition in the interior of 48-well plates and if more Petri-dishes were inoculated. Additionally there was limited change in aflatoxin production in the interior of 48-well plates if non-inoculated wells empty or filled with liquid medium. Consistent inhibition during experiments to control H_2_O suggest volatilized H_2_O was not solely responsible for the aflatoxin production inhibition. In both the 48-well plates and Petri-dishes, addition of activated charcoal did not fully restore the aflatoxin production to the highest aflatoxin level, this suggests either the adsorption of the activated charcoal was not sufficient to collect all of the inhibitory organic compound(s), or other gases are involved in the reduction in aflatoxin production.

Stimulation of aflatoxin production by increasing the number of cultures within the 48-well plates could not be attributed to any of the environmental variables manipulated. Stimulation was not eliminated by controlling pH, controlling moisture loss (by switching from liquid to solid medium or filling un-inoculated wells with liquid medium) or adsorbing volatile chemicals. Only covering with Parafilm, which presumably blocked exchange of gases between wells, prevented a stimulation of aflatoxin production. Although Parafilm could just as easily have trapped the volatile inhibitory substance. It is unlikely the stimulation was caused by changes in gases involved in respiration or large volatile organic compounds because activated charcoal readily adsorbs O_2_, CO_2_, and H_2_O and complex organic gases (Smíšek and Černý, 1967). Water is a gaseous product of respiration that could lead to stimulation in aflatoxin production, though this is inconsistent (Davis and Diener, 1968; Sanders et al., 1968; Ellis et al., 1993; Schmidt-Heydt et al., 2009). Changing to solid medium, filling the non-inoculated wells with liquid media, and activated charcoal should control moisture levels which all increased aflatoxin production. Regardless increasing the number of inoculated wells still resulted in an incremental increase of aflatoxin, making water unlikely the sole candidate for aflatoxin stimulation by greater culture density. As mentioned previously, extreme increases in CO_2_ and decreases in O_2_ both reduce aflatoxin production, so these would not directly cause the observed increases in aflatoxin production (Davis and Diener, 1968; Sanders et al., 1968; Clevström et al., 1983). Carbon dioxide at 3% compared to 0.1 and 0.7% resulted in a stimulation in aflatoxin, making it impossible to rule out CO_2_ accumulation as a candidate to stimulate aflatoxin production (Roze et al., 2004). Additionally, it is possible, as more cultures are growing within a 48-well plate, the atmospheric conditions become hypoxic and the fungus switched from aerobic respiration to anaerobic respiration and produced alcohols. Ethanol and other alcohols, including 2-ethyl-1-hexanol (produced by *A. nidulans*) and at low levels 2-buten-1-ol (produced by *A. nidulans*) have been demonstrated to stimulate aflatoxin synthesis (Reddy et al., 1979; Keller et al., 1994; Roze et al., 2007). Alcohol dehydrogenase 1 is up-regulated on conducive medium concurrent with the start of aflatoxin synthesis and throughout aflatoxin synthesis (Woloshuk and Payne, 1994). In several studies investigating volatile head space of *A. parasiticus* and *A. flavus* growing on conducive medium and corn, ethanol and other alcohols were produced (Roze et al., 2004, 2007, 2010; De Lucca et al., 2012; Spraker et al., 2014). Conceivably, 3% CO_2_ stimulates aflatoxin production because of a switch to anaerobic fermentation and ethanol caused the stimulation (Roze et al., 2004). It is likely activated charcoal did not absorb alcohols, because they are not efficiently bound by activated charcoal (Smíšek and Černý, 1967), which may explain why there was still an increase in aflatoxin production when activated charcoal was added. One explanation for lack of a substantial increase in aflatoxin production in the non-buffered L-medium is due to medium acidification (pH 1–2 after 4 days of growth). Dissolved CO_2_ from respiration should acidify medium by H^+^ disassociation from bicarbonate and other acids produced during fungal growth acidify medium (Cotty, 1988; Keller et al., 1997). Under highly acidic conditions, the hydroxyl group of alcohols can be protonated and then substituted with chlorine ions in the medium producing alkyl halides, thus functionally compromising the alcohol (Bruice, 1998).

**FIGURE 3:**
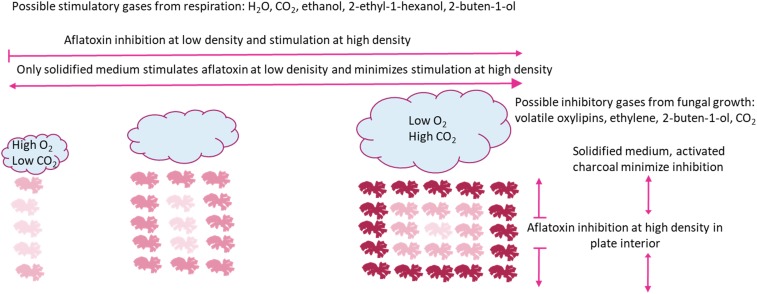
Model for aflatoxin inhibition and stimulation caused by changing density of identical *Aspergillus flavus* cultures with/in a closed system. Individual fungal mycelial cultures are represented by line drawings. Relative aflatoxin production is represented by red shade, darker red indicate greater aflatoxin production. Clouds represent gas production during fungal growth. Arrows represent aflatoxin production, blunt ends represent blocked aflatoxin production and pointed ends represent production.

We propose a model illustrated in Figure 3, where increasing well density both stimulates and inhibits aflatoxin production. The stimulation is likely a response to volatilized CO_2_ and H_2_O from respiration and alcohols during fermentation, whereas the inhibition is likely a response to greater concentrations of CO_2_ within the plate interior and volatilized oxylipins. Ecologically, inhibition of aflatoxin production at low well occupancy confounds the hypothesis that aflatoxin production is an important competitive advantage in soil environments. The lack of aflatoxin production at low “population” levels was only observed when *A. flavus* was grown in a smaller culture container (11 mm vs. 35 mm diameter), which would be closer to the expected colony size in soil. Additionally, in 24-well plates there was only a marginal increase of aflatoxin production as the number of inoculated wells increased (data not shown). A possible explanation for differential aflatoxin stimulation is energy conservation. If aflatoxin synthesis is energetically expensive, an effective conservation strategy would be to produce aflatoxin only after detection of other microorganisms. This strategy would be more important for smaller colonies, as would be expected in nature, where energy resources are more limited. Inhibition of aflatoxin production when the *A. flavus* population level becomes high, supports the hypothesis that aflatoxin is synthesized to aid in competition against other microbes and is not important if the population is well established. The inhibitory compound may be specific organic compounds related to oxylipins rather than products of respiration. Determining the volatility of oxylipins that regulate the transition from sclerotial and aflatoxin production to no toxin and conidial production deserves further review. If indeed stimulation is less specific and a response to products of respiration such as CO_2_, it stands to reason aflatoxin synthesis is switched on not only in response to other *Aspergilli*, but also other microbes and insects that are in competition for resources and potentially fungivorous.

The influence of volatiles produced by *A. flavus* on aflatoxin production warrant further attention. At this point there is evidence volatiles produced by *A. flavus* changed aflatoxin production, but no definitive information about the type of chemical cues produced by the fungus based on previous research. In the future, it will be important to test more volatiles produced by *A. flavus* to determine if these have inhibitory or stimulatory effects on aflatoxin production. It should be determined how mixtures of different volatiles effect aflatoxin to see if there are some chemicals that synergize to increase aflatoxin production or interfere with one another to inhibit aflatoxin production. Inhibitory and stimulatory volatiles could be utilized to monitor grain storage for conditions conducive to a potential aflatoxin outbreak or suggest gases to modify the storage atmospheres to be suppressive to aflatoxin production. It will be important to determine the impact *A. flavus* competition has on aflatoxin production in crops both pre and post-harvest. In spite of determining location and number of cultures affected aflatoxin production, the variability within these categories remained high, so there are more factors within an incubator leading to the variability of aflatoxin production that still needs to be understood. In the same study that found it was important to avoid excessive stacking of peanuts, location within the incubator could also affect aflatoxin production (Xue et al., 2003). Here, inoculating every well and using buffered solid medium to reduced the appearance of phenotypic variability caused by gases from neighboring colonies and is a demonstration of the need to account for and minimize the confounding effects of fungal growth when trying to understand the biology of aflatoxin production.

## Data Availability Statement

The datasets generated for this study are available on request to the corresponding author.

## Author Contributions

Both authors contributed to the conception and design of the study, manuscript revision, read, and approved the submitted version. RS conducted the experiments, organized the data, performed the statistical analysis, and wrote the manuscript.

## Conflict of Interest

The authors declare that the research was conducted in the absence of any commercial or financial relationships that could be construed as a potential conflict of interest.

## References

[B1] Abbas H. K. Weaver M. A. Horn B. W. Carbone I. Monacell J. T. Shier W. T. (2011). Selection of *Aspergillus flavus* isolates for biological control of aflatoxins in corn. *Toxin Rev.* 30 59–70. 10.1016/j.ijfoodmicro.2012.12.021 23340386

[B2] Abbas H. K. Weaver M. A. Zablotowicz R. M. Horn B. W. Shier W. T. (2005). Relationships between aflatoxin production and sclerotia formation among isolates of *Aspergillus* section *Flavi* from the Mississippi Delta. *Eur. J. Plant Pathol.* 112 283–287.

[B3] Affeldt K. J. Brodhagen M. Keller N. P. (2012). *Aspergillus* oxylipin signaling and quorum sensing pathways depend on g protein-coupled receptors. *Toxins* 4 695–717. 10.3390/toxins4090695 23105976 PMC3475224

[B4] Arai T. Ito T. Koyama Y. (1967). Antimicrobial activity of aflatoxins. *J. Bacteriol.* 93 59–64. 6020424 10.1128/jb.93.1.59-64.1967 PMC314968

[B5] Atehnkeng J. Ojiambo P. S. Donner M. Ikotun T. Sikora R. A. Cotty P. J. (2008). Distribution and toxigenicity of *Aspergillus* species isolated from maize kernels from three agro-ecological zones in Nigeria. *Int. J. Food Microbiol.* 122 74–84. 10.1016/j.ijfoodmicro.2007.11.062 18180068

[B6] Brakhage A. A. (2013). Regulation of fungal secondary metabolism. *Nat. Rev. Microbiol.* 11 21–32. 10.1038/nrmicro2916 23178386

[B7] Brown R. L. Cotty P. J. Cleveland T. E. (1991). Reduction in aflatoxin content of maize by atoxigenic strains of *Aspergillus flavus*. *J. Food Protect.* 54 623–626. 10.4315/0362-028X-54.8.623 31051605

[B8] Brown S. H. Scott J. B. Bhaheetharan J. Sharpee W. C. Milde L. Wilson R. A. (2009). Oxygenase coordination is required for morphological transition and the host-fungus interaction of *Aspergillus flavus*. *Mol. Plant Microbe Interact.* 22 882–894. 10.1094/MPMI-22-7-0882 19522570

[B9] Bruice P. Y. (1998). *Chapter 11 Reaction at an sp^3^hybridized Carbon III: Substitution and Elimination Reactions of Compounds with Leaving Groups other than Halogen Organometallic Compounds. Organic Chemistry*, 2nd Edn (Upper Saddle River: Prentice-Hall Inc), 429–433.

[B10] Buchanan R. L. Federowicz D. Stahl H. G. (1985). Activities of tricarboxylic acid cycle enzymes in aflatoxigenic strain of *Aspergillus parasiticus* after a peptone to glucose carbon shift. *Trans. Br. Mycol. Soc.* 84 267–275.

[B11] Burmeister H. R. Hesseltine C. W. (1966). Survey of the sensitivity of microorganisms to aflatoxin. *Appl. Microbiol.* 14 403–404. 5970826 10.1128/am.14.3.403-404.1966 PMC546728

[B12] Clevström G. Ljunggren H. Tegelström S. Tideman K. (1983). Production of aflatoxin by an *Aspergillus flavus* isolate cultured under a limited oxygen supply. *Appl. Environ. Microbiol.* 46 400–405. 6414371 10.1128/aem.46.2.400-405.1983 PMC239402

[B13] Cotty P. J. (1988). Aflatoxin and sclerotial production by *Aspergillus flavus*: influence of pH. *Phytopathology* 78 1250–1253.

[B14] Cotty P. J. (1989). Virulence and cultural characteristics of two *Aspergillus flavus* strains pathogenic on cotton. *Phytopathology* 79 808–814.

[B15] Cotty P. J. (1990). Effect of atoxigenic strains of *Aspergillus flavus* on aflatoxin contamination of developing cottonseed. *Plant Dis.* 74 233–235.

[B16] Davis N. D. Diener U. L. (1968). “Environmental factors affecting the production of aflatoxin,” in *Proceeding of the First U.S.-Japan Conference on Toxic Micro-Organisms*, Washington, D.C, 43–47.

[B17] De Lucca A. J. Boué S. M. Carter-Wientjes C. Bhatnagar D. (2012). Volatile profiles and aflatoxin production by toxigenic and non-toxigenic isolates of *Aspergillus flavus* grown on sterile and non-sterile cracked corn. *Ann. Agric. Environ. Med.* 19 91–98. 22462452

[B18] De Lucca A. J. Carter-Wientjes C. H. Boué S. Bhatnagar D. (2011). Volatile trans-2-hexenal, a soybean aldehyde, inhibits *Aspergillus flavus* growth and aflatoxin production in corn. *J. Food Sci.* 76 M381–M386. 10.1111/j.1750-3841.2011.02250.x 22417509

[B19] Diener U. L. Cole R. J. Sanders T. H. Payne G. A. Lee L. S. Klich M. A. (1987). Epidemiology of aflatoxin formation by *Aspergillus flavus*. *Annu. Rev. Phytopathol.* 25 249–270.

[B20] Dorner J. W. Cole R. J. Blankenship P. D. (1992). Use of a biocompetitive agent to control preharvest aflatoxin in drought stressed peanuts. *J. Food Protect.* 55 888–892. 10.4315/0362-028X-55.11.888 31084068

[B21] Doyle M. P. Marth E. H. (1978a). Aflatoxin is degraded at different temperatures and pH values by mycelia of *Aspergillus parasiticus*. *Eur. J. Appl. Microbiol. Biotechnol.* 6 95–100.

[B22] Doyle M. P. Marth E. H. (1978b). Bisulfite degrades aflatoxin: effect of citric acid and methanol and possible mechanism of degradation. *J. Food Protect.* 41 891–896. 10.4315/0362-028X-41.11.891 30812100

[B23] Drott M. T. Lazzaro B. P. Brown D. L. Carbone I. Milgroom M. G. (2017). Balancing selection for aflatoxin in *Aspergillus flavus* is maintained through interference competition with, and fungivory by insects. *Proc. R. Soc. B* 284:20172408. 10.1098/rspb.2017.2408 29263278 PMC5745424

[B24] Ellis W. O. Smith J. P. Simpson B. K. Ramaswamy H. (1993). Effect of inoculum level on aflatoxin production by *Aspergillus flavus* under modified atmosphere packaging (MAP) conditions. *Food Microbiol.* 10 525–535. 10.1016/0168-1605(94)90140-6 8074970

[B25] Fountain J. C. Scully B. T. Chen Z. Y. Gold S. E. Glenn A. E. Abbas H. K. (2015). Effects of hydrogen peroxide on different toxigenic and atoxigenic isolates of *Aspergillus flavus*. *Toxins* 7 2985–2999. 10.3390/toxins7082985 26251922 PMC4549735

[B26] Giorni P. Magan N. Peitri A. Bertuzzi T. Battilani P. (2007). Studies on *Aspergillus* section flavi isolated from maize in northern Italy. *Int. J. Food Microbiol.* 113 330–338. 17084935 10.1016/j.ijfoodmicro.2006.09.007

[B27] Horn B. W. (2003). Ecology and population biology of aflatoxigenic fungi in soil. *J. Toxicol. Toxin Rev.* 22 351–379.

[B28] Horn B. W. Gell R. M. Singh R. Sorensen R. B. Carbone I. (2016). Sexual reproduction in *Aspergillus flavus* sclerotia: acquisition of novel alleles from soil populations and uniparental mitochondrial inheritance. *PLoS One* 11:e0146169. 10.1371/journal.pone.0146169 26731416 PMC4701395

[B29] Horn B. W. Moore G. G. Carbone I. (2009). Sexual reproduction in *Aspergillus flavus*. *Mycologia* 101 423–429. 19537215 10.3852/09-011

[B30] Huang C. Jha A. Sweany R. DeRobertis C. Damann K. E. Jr. (2011). Intraspecific aflatoxin inhibition in *Aspergillus flavus* is thigmoregulated, independent of vegetative compatibility group and is strain dependent. *PLoS One* 6:e23470. 10.1371/journal.pone.0023470 21886793 PMC3158758

[B31] Joffe A. Z. Lisker N. (1969). Effect of light, temperature and pH value on aflatoxin production *in vitro*. *Appl. Microbiol.* 18 517–518. 5373682 10.1128/am.18.3.517-518.1969 PMC378018

[B32] Joshua H. (1993). Determination of aflatoxins by reversed-phase high-performance liquid chromatography with post-column in-line photochemical derivatization and fluorescence detection. *J. Chromatogr. A* 654 247–254.

[B33] Keller N. P. Butchko R. A. E. Sarr B. Phillips T. D. (1994). A visual pattern of mycotoxin production in maize kernels by *Aspergillus* spp. *Phytopathology* 84 483–488.

[B34] Keller N. P. Nesbitt C. Sarr B. Phillips T. D. Burow G. B. (1997). pH regulation of sterigmatocystin and aflatoxin biosynthesis in *Aspergillus* spp. *Phytopathology* 87 643–648. 18945083 10.1094/PHYTO.1997.87.6.643

[B35] Mehl H. L. Cotty P. J. (2010). Variation in competitive ability among isolates of *Aspergillus flavus* from different vegetative compatibility groups during maize infection. *Phytopathology* 100 150–159. 10.1094/PHYTO-100-2-0150 20055649

[B36] Niu G. Wen Z. Rupasinghe S. G. Zeng R. S. Berenbaum M. R. Schuler M. A. (2008). Aflatoxin B1 detoxification by CYP321A1 in *Helicoverpa zea*. *Arch. Insect Biochem. Physiol.* 69 32–45. 10.1002/arch.20256 18615618

[B37] Reddy T. V. Viswanathan L. Venkitasubramanian T. A. (1979). Factors affecting aflatoxin production by *Aspergillus parasiticus* in a chemically defined medium. *J. Gen. Microbiol.* 114 409–413. 541661 10.1099/00221287-114-2-409

[B38] Rohlfs M. Obmann B. (2009). Species-specific responses of dew fly larvae to mycotoxins. *Mycotoxin Res.* 25 103–112. 10.1007/s12550-009-0015-1 23604986

[B39] Roze L. V. Beaudry R. M. Arthur A. E. Calvo A. M. Linz J. E. (2007). Aspergillus volatiles regulate aflatoxin synthesis and asexual sporulation in *Aspergillus parasiticus*. *Appl. Environ. Microbiol.* 73 7268–7276. 17890344 10.1128/AEM.00801-07 PMC2168228

[B40] Roze L. V. Calvo A. M. Gunterus A. Beaudry R. Kall M. Linz J. E. (2004). Ethylene modulates development and toxin biosynthesis in *Aspergillus* possibly via an ethylene sensor-mediated signaling pathway. *J. Food Prot.* 67 438–447. 15035355 10.4315/0362-028x-67.3.438

[B41] Roze L. V. Chanda A. Laivenieks M. Beaudry R. M. Artymovich K. A. Koptina A. V. (2010). Volatile profiling reveals intracellular metabolic changed in *Aspergillus parasiticus*: *veA* regulates branched chain amino acid and ethanol metabolism. *Biomed. Central Biochem.* 11:33. 10.1186/1471-2091-11-33 PMC2939540 20735852

[B42] Roze L. V. Hong S. Y. Linz J. E. (2013). Aflatoxin biosynthesis: current frontiers. *Annu. Rev. Food Sci. Technol.* 4 293–311. 10.1146/annurev-food-083012-123702 23244396

[B43] Roze L. V. Laivenieks M. Hong S. Y. Wee J. Wong S. S. Vanos B. (2015). Aflatoxin biosynthesis is a novel source of reactive oxygen species–a potential redox signal to initiate resistance to oxidative stress? *Toxins* 7 1411–1430. 10.3390/toxins7051411 25928133 PMC4448155

[B44] Sanders T. H. Davis N. D. Diener U. L. (1968). Effect of carbon dioxide, temperature, and relative humidity of production of aflatoxin in peanuts. *J. Am. Oil Chem. Soc.* 45 683–685. 5679945 10.1007/BF02541257

[B45] Schmidt-Heydt M. Abdel-Hadi A. Magan N. Geisen R. (2009). Complex regulation of the aflatoxin biosynthesis gene cluster of *Aspergillus flavus* in relation to various combinations of water activity and temperature. *Int. J. Food Microbiol.* 135 231–237. 10.1016/j.ijfoodmicro.2009.07.026 19699547

[B46] Smíšek M. Černý S. (1967). *Chapter 4. Theory of Adsorption on Active Carbon. Active Carbon: Manufacture, Properties and Applications, English Translation* (New York, NY: American Elsevier Publishing Company, Inc.), 71–155.

[B47] Sobolev V. S. Dorner J. W. (2002). Cleanup procedure for determination of aflatoxins in major agricultural commodities by liquid chromatography. *J. AOAC Int.* 85 642–645. 12083256

[B48] Spraker J. E. Jewell K. Roze L. V. Scherf J. Ndagano D. Beaudry R. (2014). A volatile relationship: profiling an inter-kingdom dialogue between two plant pathogens, *Ralstonia solancearum* and *Aspergillus flavus*. *J. Chem. Ecol.* 40 502–513. 10.1007/s10886-014-0432-2 24801606

[B49] Sweany R. R. Damann K. E. Jr. Kaller M. D. (2011). Comparison of soil and corn kernel *Aspergillus flavus* populations: evidence for niche specialization. *Phytopathology* 101 952–959. 10.1094/PHYTO-09-10-0243 21405994

[B50] Wicklow D. T. (1991). Epidemiology of *Aspergillus flavus* in corn. *Res. Bull.* 599 315–328.

[B51] Wicklow D. T. Bobell J. R. Palmquist D. E. (2003). Effect of intraspecific competition by *Aspergillus flavus* on aflatoxin formation in suspended disc culture. *Mycol. Resour.* 107 617–723. 12884960 10.1017/s0953756203007792

[B52] Woloshuk C. P. Payne G. A. (1994). The alcohol dehydrogenase gene *adh1* is induced in *Aspergillus flavus* grown on medium conducive to aflatoxin biosynthesis. *Appl. Environ. Microbiol.* 60 670–676. 8135521 10.1128/aem.60.2.670-676.1994 PMC201364

[B53] Woloshuk C. P. Shim W. B. (2013). Aflatoxins, fumonisins, and trichothecenes: a convergence of knowledge. *FEMS Microbiol. Rev.* 37 94–109. 10.1111/1574-6976.12009 23078349

[B54] Xue H. Q. Isleib T. G. Payne G. A. Wilson R. F. Novitzky W. P. O’Brian G. (2003). Comparison of aflatoxin production in normal and high-oleic backcross-derived peanut lines. *Plant Dis.* 87 1360–1365. 10.1094/PDIS.2003.87.11.1360 30812554

[B55] Zeng R. S. Niu G. Wen Z. Schuler M. A. Berenbaum M. R. (2006). Toxicity of aflatoxin B1 to *Helicoverpa zea* and bioactivation by cytochrome P450 monooxygenases. *J. Chem. Ecol.* 32 1459–1471. 16830213 10.1007/s10886-006-9062-7

[B56] Zeng R. S. Wen Z. Niu G. Schuler M. A. Berenbaum M. R. (2007). Allelochemical induction of cytochrome P450 monooygenases and amelioration of xenobiotic toxicity in *Helicoverpa zea*. *J. Chem. Ecol.* 33 449–461. 17216359 10.1007/s10886-006-9238-1

[B57] Zeringue H. J. Jr. Bhatnagar D. Cleveland T. E. (1993). C_15_H_24_ volatile compounds unique to aflatoxigenic strains of *Aspergillus flavus*. *Appl. Environ. Microbiol.* 59 2264–2270. 16348999 10.1128/aem.59.7.2264-2270.1993 PMC182267

